# Trajectories of maternal depressive symptoms from the antenatal period to 24-months postnatal follow-up: findings from the 2015 Pelotas birth cohort

**DOI:** 10.1186/s12888-020-02533-z

**Published:** 2020-05-14

**Authors:** Nadège Jacques, Marilia Arndt Mesenburg, Alicia Matijasevich, Marlos Rodrigues Domingues, Andréa Dâmaso Bertoldi, Alan Stein, Mariangela Freitas Silveira

**Affiliations:** 1 Postgraduate Program in Epidemiology, Federal University of Pelotas, Rua Marechal Deodoro, nº 1160 3º andar, Pelotas, CEP 96020-220 Rio Grande Do Sul Brazil; 2 Departamento de Medicina Preventiva, Faculdade de Medicina FMUSP, Universidade de São Paulo, Avenida Dr. Arnaldo, 455, 2º andar, sala 2166, São Paulo, CEP 1246-903 Brazil; 3 College of Physical Education, Federal University of Pelotas, R. Luís de Camões, 625-Três Vendas, Pelotas, - RS, CEP 96055-630 Pelotas, Rio Grande Do Sul Brazil; 4 Institutional address: Department of Psychiatry, University of Oxford, Warneford Lane, Oxford, OX3 7JX UK

## Abstract

**Background:**

Maternal depression may be chronic and recurrent, with negative effects both on the health of mothers and children. Many studies have shown trajectories of postnatal depressive symptoms but few studies in low- and middle-income countries have evaluated the trajectories of depressive symptoms starting during pregnancy. This study aims to identify the different trajectories of depressive symptoms among mothers in the Pelotas 2015 birth cohort, from pregnancy to the second year of the child’s life.

**Methods:**

This study used data from the 2015 Pelotas Birth Cohort, a longitudinal study of all live births occurred in 2015 in Pelotas, Brazil. Maternal depressive symptoms were assessed using the Edinburgh Postnatal Depression Scale (EPDS). Mothers who completed the EPDS on at least three follow-up visits beginning to the antenatal follow-up visit were included in the analyses. The trajectory of maternal depressive symptoms was estimated through group-based trajectory modeling.

**Results:**

A total of 3040 women were included in the present analysis. We identified five groups of maternal depressive symptoms trajectories, with 23.4% of the mothers presenting persistent depressive symptoms and 3.9% showing chronic high depressive symptoms throughout the study period. The probability of having persistent depressive symptoms increased among mothers with greater socioeconomic vulnerability.

**Conclusions:**

This study shown the persistence of maternal depressive symptoms since pregnancy until 2 years postnatal. Additionally, alongside the known risk factors, pre-gestational depression and antenatal depressive symptoms are important risk factors for the persistence and severity of depressive symptoms. These findings support the need to provide mental health evaluation and care for women from pregnancy to the late postnatal period.

## Background

Depression is a mental disorder common among women in antenatal and postnatal periods due to biological, emotional, psychological, social and cultural changes experienced at this time [1–7]. A systematic review of longitudinal studies on antenatal and at one-year postpartum depression indicated a mean prevalence of 17 and 13%, respectively [8]. Antenatal depression is a major predictor of postnatal depression [9–11], with other associated factors: living alone, having more than two children, low education, and unemployment [8, 12]. Maternal depressive symptoms may be chronic or recurrent, and have major consequences for the health of mothers and children [8, 13–15].

Trajectory analysis has been used in psychology to understand etiology and developmental course of different types of disorders including depression [16]. Few studies have analyzed trajectories of maternal depression including pre- and postnatal periods, and some study specific low income populations within high-income countries [17] but none in low- or middle-income countries This is consistent with the research gap on the trajectories of depression in low- and middle-income countries, which may be different [18, 19].

The present study aims to identify depression symptoms trajectories and associated risk factors among mothers of participants from the 2015 Pelotas Birth Cohort (2015 Cohort), from the antenatal period through the second year of offspring’s life.

## Methods

### Participants

The 2015 Cohort, a longitudinal study of all live births, occurred in Pelotas, a midsized city in southern Brazil, being the most recent of four cohorts conducted in the city [20]. Unlike previous cohorts, this one recruited mothers during pregnancy. All mothers expected to deliver in 2015 were identified through an active search strategy. For the perinatal follow-up visit, interviews were performed 24 to 48 h after delivery. For 3-and 12-month follow-up, the mothers were interviewed at home; for 24-month follow-up, the interviews were performed at the research center. More information available at the 2015 Cohort profile [20].

### Measures

#### Maternal depressive symptoms

Maternal depressive symptoms were measured during antenatal, 3-, 12-, and 24-month visits, using the Edinburgh Postnatal Depression Scale (EPDS) [21–24]. EPDS is comprised of 10 items, each coded from 0 to 3 [21] being validated for use in Brazil, using the ≥10 as the best cutoff for this population to identify mothers with depressive symptoms [25].

### Covariates

The following information was collected during the perinatal interview: Brazilian economic classification criterion (ABEP), measured by ownership of household goods (continuous variable); maternal education (complete years of formal education); maternal age (whole years); marital status (living partner – yes/no); maternal skin color (white/black or brown); parity (number of previous deliveries, including stillbirths – < 2 / ≥2); planned pregnancy (yes/no); number of antenatal consultations (< 6 / ≥ 6); work during pregnancy (yes/no); smoke during pregnancy (smoked at least one cigarette daily in any trimester of pregnancy – yes/no); consumption of alcohol during pregnancy (any amount of alcohol intake during any trimester of pregnancy – yes/no); use of illicit drugs during pregnancy (yes/no); pre-gestational depression (yes/no); type of delivery (vaginal/cesarean); and self-assessment of health status (excellent/good, poor/very poor).

Child variables included: sex (male or female); birth weight (< 2500 g, low; ≥ 2500 g, normal); and 5-min Apgar score (≤ 6 or ≥ 7). Mothers were asked whether the newborn needed admission to an intensive care or high-dependency unit (yes/no), classified as “rooming-in” or “intensive or intermediate care”.

### Statistical analyses

All mothers of 2015 Cohort participants who completed EPDS on at least three follow-up visits (one of which, necessarily, the antenatal follow-up visit) were included in the analyses.

Characteristics of the mother–child pairs were described. Sensitivity analysis was performed comparing the characteristics of the mothers and children included in the present analysis with all 2015 Cohort participants. The maternal depressive symptoms trajectory was estimated through group-based trajectory modeling. This semi-parametric method proposed by Nagin & Tramblay [26] and Nagin [27] aims to identify groups of individuals that develop a similar trajectory by evaluating the outcome of interest on at least three time points. Missing data were retained in the analysis and handled by the model through maximum likelihood estimation.

Approximately 25% of mothers presented an EPDS score ≤ 4 at each follow-up, indicating a non-normal distribution. A polynomial function model was used, assuming a censored normal (c norm) distribution, designed for analysis of approximately continuous scales, measured repeatedly, that can be censored by a minimum or maximum scale or both (e.g., longitudinal data on a depressive symptoms scale) [28]. The model facilitates disclosure of the relationship between maternal EPDS score, gestational age, and child age, using age for time indexing [16, 26, 27]. Models were estimated using the “traj” command in Stata [28].

Number and shape of the trajectories were chosen based not only on best model fit, evaluated through the maximum Bayesian information criterion (BIC), but also on the interpretability of the trajectories obtained [27]. Selection of the appropriate model was guided by posterior probability scores for each trajectory group. Those with a mean probability score > 0.7 were selected for all groups.

Contribution of each predictive variable was examined. Each trajectory group was compared in terms of maternal and child characteristics through analysis of variance (ANOVA) for continuous variables and the chi-square test for dichotomous variables.

## Results

All 4387 children from the urban area, born in hospitals in Pelotas (> 99% of births) between January 1 and December 31, 2015 were eligible for inclusion. Of all mothers, 4275 (98.7%) agreed to participate. The response rates at the follow-up visits were 97.2% (3 months), 95.4% (12 months), and 95% (24 months). Mothers of 73.8% of all participants were interviewed during pregnancy [20]. Of these mothers, 3900 (91%) attended at least three follow-up visits, with 3040 (78%) having completed EPDS during antenatal care and in at least two other follow-up visits, being included in the present analysis.

Table 1 describes the characteristics of the sample included in this study and a comparison with the entire 2015 Cohort. Mothers included in this study had higher average income, education, and age; higher proportions of white mothers and cesarean delivery; lower proportions of mothers living alone, with two or more children, unplanned pregnancy, six or fewer antenatal consultations, and who worked, smoked, or consumed alcohol during pregnancy. Children of mothers included in the analyses had lower rates of low birth weight, 5-min Apgar score ≤ 6, and need for high-dependency or intensive care unit admission.

**Table 1:** Comparison of maternal and child characteristics between those included and not included in the present study

Characteristics	Included (***n*** = 3040)	Not included (***n*** = 1235)	***p***-value
**Maternal**			
ABEP, mean (sd)*a*	25.7 (9.4)	23.6 (10.4)	< 0.001
Schooling (years), mean (sd)*a*	10.4 (3.8)	9.1 (4.2)	< 0.001
Maternal age (years), mean (sd)*a*	27.4 (6.5)	26.5 (6.8)	< 0.001
Single mother, (%)	12.6	18.0	< 0.001
Skin color, White (%)	73.2	66.6	< 0.001
Parity ≥2 (%)	48.8	55.1	< 0.001
Depression before pregnancy (%)	17.2	16.4	0.784
Unplanned pregnancy (%)	42..8	53.4	< 0.001
Prenatal consultations ≤6 (%)	10.5	24.4	< 0.001
Worked during pregnancy (%)	41.7	50.7	< 0.001
Smoking during pregnancy (%)	13.8	23..1	< 0.001
Alcohol during pregnancy (%)	6.8	8.8	0.021
Drugs during pregnancy (%)	1.2	0.0	0.290
C-section (%)	67.0	60.7	< 0.001
Self-rated health poor/very poor (%)	13.7	13.0	0.623
**Child**			
Sex, male (%)	50.7	50.4	0.831
Low birthweight (%)	9.3	11.9	0.008
APGAR 5′ ≤ 6 (%)	0.8	2.4	< 0.001
Intermediate or intensive care hospitalization after birth (%)	7.7	10.4	0.005

X^2^ Test for all except *a*

*a* ANOVA test

### Trajectories of maternal depressive symptoms

Analysis of the trajectories of maternal depressive symptoms, at four time points (antenatal period and 3, 12, and 24 months after delivery), showed that the five-group model was more adequate and parsimonious than the less or more group model, as it better demonstrated the heterogeneity between groups presenting the best model fit, within the recommended lower limit of posterior probability of 0.7 for each group (Table 2). Three trajectories were best represented by a cubic term, one trajectory by a quadratic term, and one by a linear term (Table 2).

**Table 2:** Descriptive and average posterior probabilities (APP) for the trajectories (n = 3040)

Trajectories of depressive symptoms	N (%)	APP (sd)	Parameters	Parameter Estimates		
				B	(se)	p-value
1 “Low” depressive symptoms	1109 (36.5)	0.84 (0.14)	Intercept	3.210	(0.148)	< 0.001
			Linear	−0.085	(0.011)	< 0.001
			Quadratic	0.003	(0.000)	< 0.001
2 ‘Moderate low” depressive symptoms	1219 (40.1)	0.78 (0.14)	Intercept	5.687	(0.196)	< 0.001
			Linear	−0.068	(0.013)	< 0.001
			Quadratic	0.024	(0.002)	< 0.001
			Cubic	−0.000	(0.000)	< 0.001
3 “Increasing” depressive symptoms	298 (9.8)	0.73 (0.17)	Intercept	10.232	(0.375)	< 0.001
			Linear	0.155	(0.021)	< 0.00
4 “Decreasing-persistent” depressive symptoms	294 (9.7)	0.72 (0.17)	Intercept	11.248	(0.392)	< 0.001
			Linear	−0.381	(0.052)	< 0.001
			Quadratic	0.040	(0.006)	< 0.001
			Cubic	−0.001	(0.000)	< 0.001
5 “High chronic” depressive symptoms	120 (3.9)	0.90 (0.15)	Intercept	15.911	(0.379)	< 0.001
			Linear	−0.033	(0.042)	0.432
			Quadratic	0.040	(0.007)	< 0.001
			Cubic	−0.001	(0.000)	< 0.001

**Fig. 1:**
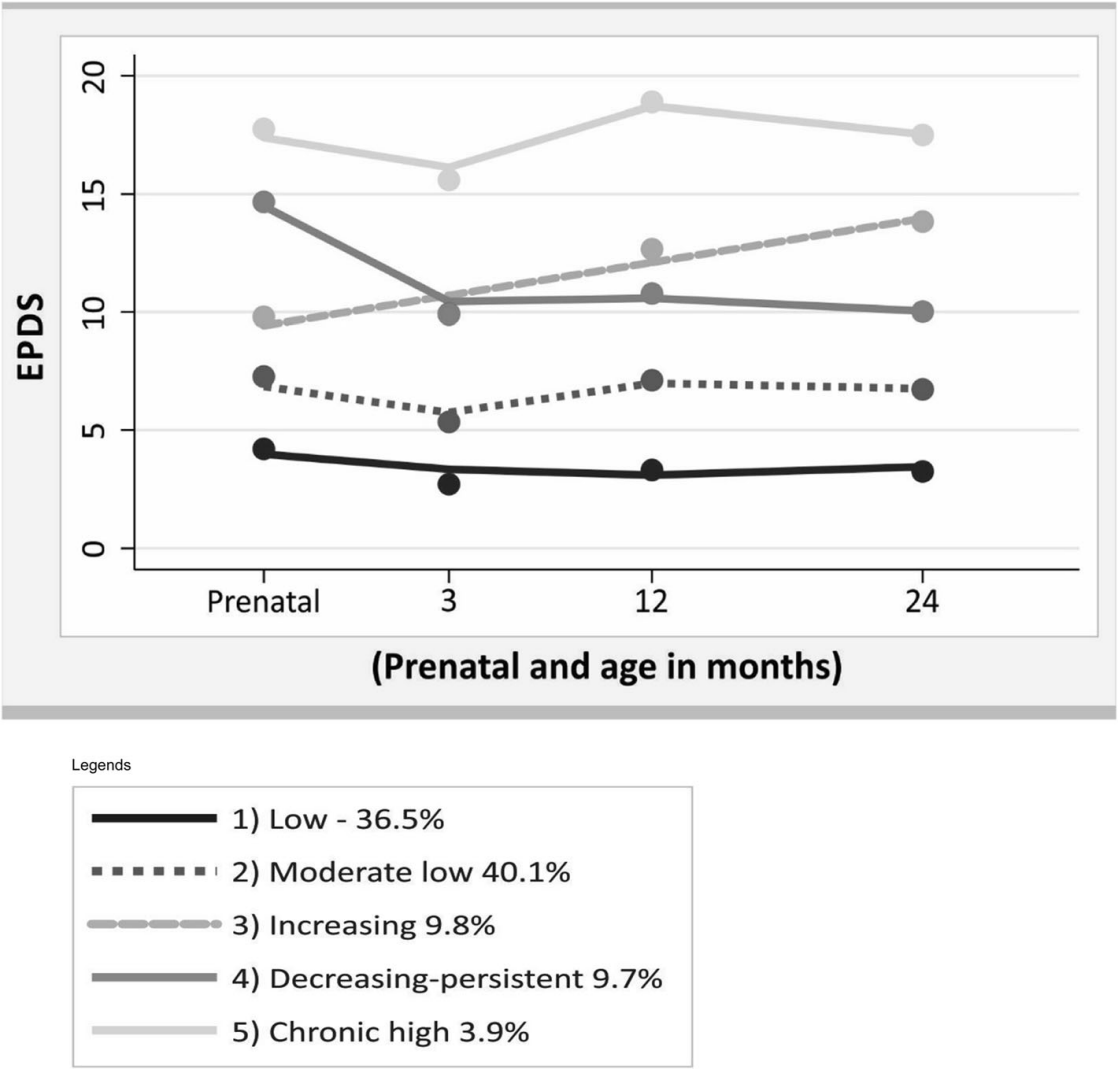
Trajectories of maternal depressive symptoms prenatal and child’s age, Pelotas Birth Cohort 2015, RS, Brazil

The first (“Low” depressive symptoms) and the second (“Moderate low” depressive symptoms) groups had an EPDS score < 10 at all-time points of analysis and included 76.6% of the mothers. The third group (“Increasing” depressive symptoms) included 9.8% of the mothers, who exhibited a constant increase in EPDS scores over the period of analysis. The fourth group (“Decreasing but persistent” depressive symptoms) included 9.7% of the mothers, who presented a high depressive symptom score in the antenatal period, had a decrease at 3 months, and maintained a score around 10 between the 12- and 24-month time points. Finally, the fifth group (“Chronic high” depressive symptoms), which comprised 3.9% of the mothers, presented a high EPDS scores of 17 in the antenatal period, declining to 15 at 3 months, rising again to 19 at 12 months, and again decreasing to 17 at 24 months after delivery (Fig. 1).

### Factors associated with trajectories of maternal depressive symptoms

Trajectories of maternal depressive symptoms were associated with maternal but not with offspring characteristics. The “increasing”, “decreasing but persistent”, and “chronic high” groups accounted for a higher proportion of unmarried mothers, with two or more children, with history of pre-gestational depression, unplanned pregnancies, who attended six antenatal visits or fewer, self-assessed their health as poor or very poor, and who worked, smoked, and drank alcohol during pregnancy. Those groups had a lower proportion of white women, with low educational attainment, and who had a cesarean delivery compared with “low” and “moderate low” trajectory groups (Table 3).

**Table 3:** Maternal and child characteristics according to maternal depressive symptoms trajectories groups

Variables	Maternal depressive symptoms trajectories groups					*p*-Value
	Group 1 “Low”	Group 2 “Moderate low”	Group 3 “increasing”	Group 4 “Decreasing-persistent”	Group 5 “Chronic high”	
	***n*** = 1109	***n*** = 1219	***n*** = 298	***n*** = 294	***n*** = 120	
**Maternal variables**						
ABEP, mean (sd)*a*	28.0 (9.6)	25.8 (9.0)	23.5 (8.8)	21.2 (8.3)	18.8 (6.3)	< 0.001
Schooling (years), mean (sd)*a*	11.6 (3.7)	10.5 (3.7)	9.3 (3.7)	8.2 (3.6)	7.3 (2.9)	< 0.001
Maternal age (years), mean (sd)*a*	28.1 (6.2)	27.1 (6.6)	27.4 (6.9)	25.8 (6.6)	27.0 (6.9)	< 0.001
Single mother, (%)	7.6	12.9	13.8	23.8	25.8	< 0.001
Skin color, White (%)	79.1	74.0	68.6	57.6	60.0	< 0.001
Parity ≥2 (%)	42.7	46.1	60.3	60.8	73.3	< 0.001
Depression before pregnancy (%)	8.5	16.1	24.2	33.3	51.7	< 0.001
Unplanned pregnancy (%)	36.1	41.7	50.5	54.76	66.7	< 0.001
Prenatal consultations ≤6 (%)	7.3	8.9	13..5	17.6	21.9	< 0.001
Worked during pregnancy (%)	35.3	40.1	53.2	54.1	59.2	< 0.001
Smoking during pregnancy (%)	6.8	13.9	21.5	27.5	25.8	< 0.001
Alcohol during pregnancy (%)	4.9	5.9	11.1	11.2	11.7	< 0.001
Drugs during pregnancy (%)	0.3	0.9	2.7	3.4	2.5	< 0.001
C-section (%)	70.6	67.1	63.9	60.5	55.8	< 0.001
Self-rated health poor/very poor (%)	4.5	11.9	25.6	24.9	60.7	< 0.001
**Child variables**						
Sex, male (%)	51.5	49.3	54.0	51.4	48.3	0.580
Low birthweight (%)	9.9	8.9	7.4	9.5	11.7	0.574
APGAR 5′ ≤ 6(%)	0.9	1.0	0.0	0.3	1.7	0.302
Intermediate or intensive care hospitalization after birth (%)	7.9	7.4	6.7	7.8	11.7	0.510

X^2^ Test for all except *a*

*a* ANOVA test

Mothers in the “low” depressive symptoms group had higher: socioeconomic status, education level, proportion of white mothers, average age and prevalence of cesarean delivery. Mothers in the “chronic high” depressive symptoms group had lower socioeconomic level, lower educational attainment, higher probability of not living with a partner, having two or more children, unplanned pregnancy, history of pre-gestational depression, attended fewer than six antenatal consultations, worked during pregnancy, and self-assessing their health as poor. There was no difference in mean age among the “moderate low”, “increasing”, and “chronic high” groups. The “decreasing but persistent” group had a higher prevalence of mothers who smoked and used drugs during pregnancy. There was no difference in alcohol intake between the “increasing”, “decreasing but persistent”, and “chronic high” groups (Table 3).

## Discussion

Our main finding was the identification of five groups of maternal depressive symptoms trajectories, from the antenatal period up to 2 years after birth. Three-quarters of the mothers had “low” and “moderate low” trajectories, a mild level of depressive symptoms throughout the period. “Increasing” depressive symptoms group presented EPDS scores of 13 at 24 months postpartum and lower than 13 at the earlier assessments, indicating probable postnatal depression at the end of the period. “Decreasing but persistent” depressive symptoms group presented depressive symptoms throughout the study period, with a decrease after pregnancy (EPDS score 14 at antenatal period, decreasing to 10 at 3 months, and remaining within this range until 24 months postpartum).

We also identified a group of women with high scores of EPDS throughout the study period. Although this group was small (3.9%), it is a high-risk group from a mental health standpoint due to the lack of improvement over time.

A study conducted with the 2004 Pelotas Birth Cohort evaluated trajectories of postnatal maternal depression from three months to 6 years of age [13], using the same EPDS cutoff point used in our study, with five trajectories identified. The “Low” and “moderate low” depressive symptom groups are comparable to the same categories in our study, however, results for the “increasing”, “decreasing”, and “high” groups differed from ours. The “increasing” group in 2004 showed no symptoms of maternal depression in the first 18 months after delivery, but EPDS scores increased thereafter, up to 15 at 60 months postpartum. “Decreasing” group in 2004 presented an EPDS score of 12 at the start of the study period, decreasing to nine at the end, indicating mild symptoms of postnatal depression at the end. The “high” depressive symptoms group in 2004 presented an upward curve, with scores from 15 to 19 at the end of the period. This allows the identification of a group of women with a high likelihood of developing postnatal depression. These differences might be explained by differences in the periods evaluated and in the age of the offspring. The present study, despite a shorter period of analysis, brings important supplementary information, such as symptoms of antenatal depression, which transforms the trajectory, indicating that the maternal depressive symptoms, when present since the antenatal period, could persist in the late postnatal period. Recent literature review indicates that 6.6% of women experience persistent depression from antenatal and postnatal periods [8].

A maternal depression trajectories study in the US included primiparous women (16–21 years of age) from a randomized controlled longitudinal study [29], in which the presence of depressive symptoms was evaluated antenatally, at 12 and 24 months. The authors used latent growth curve modeling (LGCM) to identify trajectories and the Center for Epidemiological Studies-Depression (CES-D) scale, with a cutoff point of 16, to evaluate clinical symptoms of depression. Mothers were divided into two groups (with vs. without antenatal symptoms of depression), and trajectories were evaluated separately for each group. Both groups received a support intervention through a home-visit program, which may have reduced the prevalence of depressive symptoms. Among women with depressive symptoms during the antenatal period, there was a downward trend, and those who were not antenatally depressed reported a slight increase in symptoms over the 2-year study period, which indicates that some women developed symptoms of postpartum depression. Differences between these results and the present investigation may be attributable to different methods of trajectory analysis and evaluation of depressive symptoms. Furthermore, only adolescent and young adult mothers were included in the study, and young age is a known predictor of antenatal and postnatal depression.

We identified two other studies that used group-based trajectory modeling and included the antenatal period: Luoma et al. [18] evaluated depressive symptoms trajectories in 325 primiparous women in the third trimester of pregnancy in Finland. EPDS was used to assess symptoms of depression from the antenatal period up to 16–17 years of age, at six points in time (pre-partum, 2 months, 6 months, 4–5 years, 8–9 years, and 16–17 years postpartum). Four groups of trajectories were identified: “intermittent” group (moderate symptoms of antenatal depression and significant symptoms of depression at 4–5 years postnatal and during the child’s adolescence); “high-stable” group (mothers who remained within the normal range with a low EPDS cutoff point); and “low-stable” and “very-low” groups (median EPDS scores well below the conventional cutoff point for depression) [18]. Differences between our results might be explained by the longer period of analysis. Pre-adolescence and adolescence pose many new challenges for mothers, and may influence the development of maternal depression. Different levels of depressive symptoms in the studied population and smaller sample size also may have influenced the number of groups identified and the direction of trajectories. Another study conducted in Netherlands [19]. with 4167 pregnant women, using the self-administered Brief Symptom Inventory (BSI) to screen for depressive symptoms [19], identified four trajectories: “no” trajectory (34%), “low” trajectory (54%), “moderate” trajectory (11%), and “high” trajectory (1.5%). Compared to the present investigation, these two studies did not find ascending or descending trajectories; probably due to the types of population studied.

Giallo et al. [14] examined the postnatal depressive symptoms trajectories in 4879 women in Australia, at four time points. Results indicated a steady rise in the development of depressive symptoms from 3 months to 7 years postpartum, with prevalence of depressive symptoms ranging from 7 to 11%. Two groups of latent symptoms were identified: “minimal depressive symptoms” (some symptoms in the first postnatal year that decreased over time), and “persistently high depressive symptoms” (symptoms persisted and increased slightly over time) [14]. Our study found persistence of maternal depressive symptoms in three of the five trajectory groups.

Despite being similar overall, there are some differences in the patterns and numbers of trajectories found among the studies. This might be explained by factors such as the study’s methodology, the target population, the period studied, the instrument used to measure the depressive symptoms and the cut-off used. Further, by personal factors such as socio-economic status, physical and mental health indicators, pregnancy-related factors, health habits. These personal factors do not differ between mothers with depressive symptoms living in high income or low- and middle-income countries, but might influence the patterns of the trajectories between countries [14, 18].

With respect to risk factors, the likelihood of belonging to “increasing”, “decreasing but persistent”, and “chronic high” groups was higher among mothers with characteristics commonly associated with maternal depression, such as poverty, low educational attainment, not living with a partner, having more children, unplanned pregnancies, fewer antenatal consultations, working, smoking, and using alcohol or drugs during pregnancy, and having a poor self-perception of health. These findings are consistent with literature, regardless of the depression-screening tool used and the country in which the study was conducted [30–36]. Our findings corroborate those of Cents that the likelihood of belonging to moderate and high depressive-symptom trajectories increased with the presence of these risk factors [19].

The “chronic high” group was not only associated with all predictors of antenatal and postnatal depression, but also had a higher proportion of women with pre-gestational depression and poor health self-reported. This is consistent with other studies that found a higher likelihood of belonging to the group with the “higher” depression trajectory for mothers with history of pre-gestational depression [19, 32, 33, 37].

We found no association between any of the maternal depressive symptoms trajectory groups and offspring characteristics, in contrast to a previous study from Pelotas [13] that found an association with male offspring and prematurity, as other studies [38]. This difference maybe could be explained by the inclusion of antenatal depressive symptoms in the trajectories.

### Limitations and strengths of the study

This study has some limitations. First, not all mothers participating in the cohort were included in the analysis, with the mothers not included presenting worse sociodemographic and health conditions, characteristics often associated with symptoms of depression. This may have led to an underestimation of the proportion of mothers with depressive symptoms.

Another limitation is that the EPDS was not administered antenatally at the same gestational age for all women, preventing classification of the trajectory of depressive symptoms during pregnancy by trimester.

Few mothers reported illicit drug use during pregnancy, which may indicate self-censorship in face of perceived inappropriate behavior. Although most mothers reporting illicit drug use during pregnancy were classified into the “decreasing but persistent” depressive symptoms trajectory, the underestimation of this use may have prevented assessment of the impact of this behavior on depressive symptoms. A history of psychiatric disorders, anxiety disorders and treated mothers are not available data, so we were not able to compare them or predict their trajectories.

The strengths of the study include the fact that this was the first population-based study of maternal depression trajectories conducted in a low- or middle-income country that includes gestational depression. Moreover, the occurrence of depressive symptoms was measured longitudinally from the antenatal period to 24 months postpartum, using a validated instrument.

## Conclusion

More than 23% of mothers from the Pelotas 2015 Birth Cohort experienced persistent depressive symptoms from pregnancy to 2 years postpartum. It is important to highlight that the “increasing”, “decreasing but persistent”, and “high chronic” groups constitute the persistent groups with different severity levels, which were found between an important transition period. These findings suggest that there may be several groups, patterns, or trends in trajectories among those with depressive symptoms and that alongside the known risk factors, pre-gestational depression and antenatal depressive symptoms are important risk factors for the persistence and severity of depressive symptoms. Therefore, it is necessary to include screening for depressive symptoms not only at the immediate postpartum period, but also throughout pregnancy and in late postnatal periods, ensuring identification and treatment of at-risk women.

## Data Availability

The data that support the findings of this study are available from the 2015 Pelotas (Brazil) Birth Cohort, but restrictions apply to the availability of these data, which were used under license for the current study, and so are not publicly available. Data are however available from the “Centro de Pesquisas Epidemiológicas (CPE) of Pelotas, RS, Brazil” on request by the publications committee upon reasonable request at [cpublicacoes.coortespelotas@gmail.com].

## Abbreviations

ABEP: Brazilian Economic Classification Criteria
APGAR: The Apgar Score is a method to quickly summarize the health of newborn children against infant mortality
APP: Average Posterior Probabilities
BIC: Bayesian Information Criterion
EPDS: Edinburgh Postnatal Depression Scale
